# Meiosis in allopolyploid *Arabidopsis suecica*

**DOI:** 10.1111/tpj.15879

**Published:** 2022-07-22

**Authors:** Candida Nibau, Adrián Gonzalo, Aled Evans, William Sweet‐Jones, Dylan Phillips, Andrew Lloyd

**Affiliations:** ^1^ Institute of Biological, Environmental & Rural Sciences (IBERS) Aberystwyth University Penglais Aberystwyth Ceredigion SY23 3DA UK; ^2^ John Innes Centre Colney Lane Norwich NR4 7UH UK; ^3^ Department of Biology, Institute of Molecular Plant Biology Swiss Federal Institute of Technology (ETH) Zürich Zürich 8092 Switzerland

**Keywords:** *Arabidopsis suecica*, meiosis, polyploid, polyploidy, recombination, crossover, homoeologous recombination, whole‐genome duplication

## Abstract

Polyploidy is a major force shaping eukaryote evolution but poses challenges for meiotic chromosome segregation. As a result, first‐generation polyploids often suffer from more meiotic errors and lower fertility than established wild polyploid populations. How established polyploids adapt their meiotic behaviour to ensure genome stability and accurate chromosome segregation remains an active research question. We present here a cytological description of meiosis in the model allopolyploid species *Arabidopsis suecica* (2*n* = 4*x* = 26). In large part meiosis in *A*. *suecica* is diploid‐like, with normal synaptic progression and no evidence of synaptic partner exchanges. Some abnormalities were seen at low frequency, including univalents at metaphase I, anaphase bridges and aneuploidy at metaphase II; however, we saw no evidence of crossover formation occurring between non‐homologous chromosomes. The crossover number in *A*. *suecica* is similar to the combined number reported from its diploid parents *Arabidopsis thaliana* (2*n* = 2*x* = 10) and *Arabidopsis arenosa* (2*n* = 2*x* = 16), with an average of approximately 1.75 crossovers per chromosome pair. This contrasts with naturally evolved autotetraploid *A*. *arenosa*, where accurate chromosome segregation is achieved by restricting crossovers to approximately 1 per chromosome pair. Although an autotetraploid donor is hypothesized to have contributed the *A*. *arenosa* subgenome to *A*. *suecica*, *A*. *suecica* harbours diploid *A*. *arenosa* variants of key meiotic genes. These multiple lines of evidence suggest that meiosis in the recently evolved allopolyploid *A*. *suecica* is essentially diploid like, with meiotic adaptation following a very different trajectory to that described for autotetraploid *A*. *arenosa*.

## Significance Statement

In this manuscript we describe meiosis in the allopolyploid model species Arabidopsis suecica, showing that meiosis, and the genes controlling it, are essentially diploid‐like. Meiotic adaptation in allopolyploid A. suecica therefore follows a very different route to that previously described for autotetraploid A. arenosa.

## INTRODUCTION

All flowering plants have undergone multiple rounds of whole‐genome duplication (WGD) in their evolutionary past (Jiao et al., 2011; Soltis et al., 2015; Van de Peer et al., 2009), and around 25–30% of all angiosperms are thought to be recently formed polyploids (Barker et al., 2016; Wood et al., 2009). Although polyploidy is common, there are several obstacles that a new polyploid must overcome to become established. One of these is the difficulty of accurately segregating multiple related or identical sets of chromosomes during meiosis. As a result, first‐generation polyploids often have low fertility associated with chromosome segregation errors and genomic rearrangements (Darlington, 1929; Grandont et al., 2013; Lloyd & Bomblies, 2016; McCollum, 1958; Szadkowski et al., 2010; Yant et al., 2013). How the challenge of accurate chromosome segregation is met depends in large part on the nature of the polyploidy event (Lloyd & Bomblies, 2016).

As a useful generalization, albeit an acknowledged simplification, polyploids fall into two categories: autopolyploids and allopolyploids (Ramsey & Schemske, 2002). Autopolyploids arise from within‐species WGD and so have multiple equally divergent copies of each chromosome. In contrast, allopolyploids like *Arabidopsis suecica* arise from WGD associated with interspecific hybridization, and thus have two distinct subgenomes (Burns et al., 2021; Jiang et al., 2021; O'Kane et al., 1996). In allopolyploids, each chromosome therefore has one true homologue belonging to the same subgenome, as well as multiple divergent (but still closely related) homoeologous chromosomes belonging to the other subgenome(s).

The challenges of chromosome segregation differ for autopolyploids and allopolyploids. In allopolyploids there must be preferential crossover formation between true homologues and the suppression of crossovers between homoeologous chromosomes. This ensures regular homologous bivalent formation and accurate chromosome segregation. In autopolyploids, accurate chromosome segregation must be accomplished without any preferential bivalent formation. Recent studies in autopolyploid Arabidopsis indicate that one adaptive strategy to ensure faithful chromosome segregation is to restrict recombination, via ‘supercharged’ crossover interference so that each chromosome only forms one crossover (Morgan et al., 2021). This ensures only bivalents occur at metaphase I, resulting in accurate chromosome segregation without preferential bivalent formation. Several genes associated with the chromosome axis are implicated in this adaptation (Bohutínská et al., 2021; Seear et al., 2020; Wright et al., 2015; Yant et al., 2013).

Despite many decades of research, the factors that promote meiotic stability in allopolyploids are only just beginning to come to light. Perhaps the most significant of recent discoveries is the identification of the gene within the *Ph1* locus in *Triticum aestivum* (wheat) responsible for suppressing homoeologous recombination. CRISPR mutagenesis demonstrated that *TaZIP4‐B2*, an additional copy of *ZIP4* within the *Ph1* locus on chromosome 5B (three syntenic copies of *ZIP4* are also present on chromosomes 3A, 3B and 3D), is responsible for the suppression of homoeologous crossovers in wheat and wheat hybrids (Martín et al., 2021; Rey et al., 2017). ZIP4 belongs to the ZMM group of proteins that bind to and stabilize meiotic recombination intermediates (Chelysheva et al., 2007; Lynn et al., 2007; Tsubouchi et al., 2006). It acts as a scaffold (via tetratricopeptide repeats) for the formation of multiprotein complexes and couples meiotic crossover formation to the assembly of the synaptonemal complex (De Muyt et al., 2018; Pyatnitskaya et al., 2022). Another ZMM protein has also recently been shown to influence the stringency of meiotic recombination. In *Brassica napus*, reducing the copy number of *MSH4* decreases the recombination occurring between divergent homoeologous chromosomes but has no effect on the number of crossovers occurring between homologous chromosomes (Gonzalo et al., 2019), i.e. reducing the functional dosage of *MSH4* increases the stringency of meiotic recombination. Taken together, these two findings suggest that recombination intermediate stability may be a major factor influencing the stringency of meiotic recombination and the suppression of homoeologous crossovers.

The other pathway known to play a role in determining the stringency of recombination is the mismatch repair (MMR) pathway (Spies & Fishel, 2015). The exact nature of this role, and whether it differs between meiotic and somatic recombination has, however, been hard to pin down. There is convincing evidence that the universal MMR subunit MSH2 suppresses meiotic recombination in yeast hybrids (Bozdag et al., 2021). In plants the evidence had been somewhat more equivocal, with MSH2 proposed to both suppress (Tam et al., 2011) and promote (Blackwell et al., 2020) meiotic recombination between divergent sequences. Recently, however, a copy of *MSH7* on chromosome 3D of wheat was identified as *Ph2*, a suppressor of homoeologous recombination (Serra et al., 2021), suggesting that this gene at least promotes increased stringency of meiotic recombination.

The model allopolyploid *A*. *suecica* (2*n* = 2*x* = 26) has one subgenome donated by the diploid model species *Arabidopsis thaliana* (2*n* = 2*x* = 10) and the other subgenome donated by *Arabidopsis arenosa. Arabidopsis arenosa* occurs as both diploid (2*n* = 2*x* = 16) and autotetraploid (2*n* = 4*x* = 32) cytotypes (Burns et al., 2021; Jiang et al., 2021; O'Kane et al., 1996), with an autotetraploid considered the most likely parent of *A*. *suecica* (Novikova et al., 2017). Like many allopolyploids, natural populations of *A*. *suecica* are highly fertile but first‐generation synthetic or ‘neo’ tetraploids have very low pollen viability, associated with increased meiotic errors (Henry et al., 2014). Using an F_2_ population derived from a cross between natural and synthetic *A*. *suecica*, Henry et al. (2014) identified a quantitative trait locus (QTL) on chromosome 4 of the *A*. *arenosa* subgenome associated with high pollen viability, although the nature of any underlying gene is unknown. Recent publications have identified numerous genes with altered expression patterns in natural *A*. *suecica*, compared with its diploid progenitors (Burns et al., 2021; Jiang et al., 2021). Genes with higher expression in natural *A*. *suecica* include many involved in chromosome integrity and structural maintenance, such as the cohesins *SMC1* and *SMC3*, cohesion cofactors *PDS5A*–*PDS5D*, members of the SMC5/6 complex (i.e. *SMC5* and *SMC6B*), RECQ family helicases (*RECQL1*–*RECQL3*) and components of the MCM helicase (*MCM2*–*MCM6*) (Burns et al., 2021; Jiang et al., 2021). In at least some cases, increased expression is likely to result from reduced gene‐body methylation (Jiang et al., 2021). Although many of these genes have roles in meiosis, the observed differences in gene expression may well reflect altered requirements for chromosome maintenance and replication during somatic growth, particularly given that leaf tissue was used in the analyses (Burns et al., 2021; Jiang et al., 2021). Whether any of these genes also have altered meiotic expression remains to be seen.

Over the last two decades, the *Arabidopsis* genus has emerged as a powerful model for meiosis research (Mercier et al., 2014) and for investigations of biological processes in polyploids (Bomblies & Madlung, 2014). With the recent publication of two high quality *de novo* genome assemblies (Burns et al., 2021; Jiang et al., 2021), and the emergence of highly efficient plant gene editing (Grützner et al., 2021), the model allopolyploid *A*. *suecica* will be a powerful tool for functional studies of allopolyploid meiosis. We present here a characterization of wild‐type meiosis in *A*. *suecica* that will serve as a resource for future investigations of allopolyploid meiosis in this emerging model.

## RESULTS

### *Arabidopsis suecica* shows a largely diploid‐like meiosis, although some abnormalities are observed

**Figure 1:**
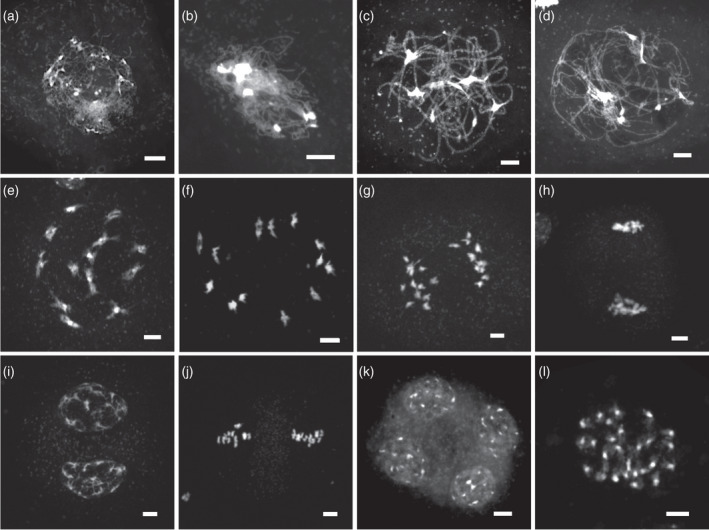
Meiotic progression in *Arabidopsis suecica*. DAPI‐stained meiotic cell nuclei: (a) leptotene; (b) early zygotene; (c) pachytene; (d) early diplotene; (e) diakinesis; (f) metaphase I; (g) anaphase I; (h) telophase I; (i) dyad; (j) metaphase II; (k) tetrad; (l) somatic nucleus showing 26 distinct chromosomes. Scale bars: 5 μm.

**Figure 2:**
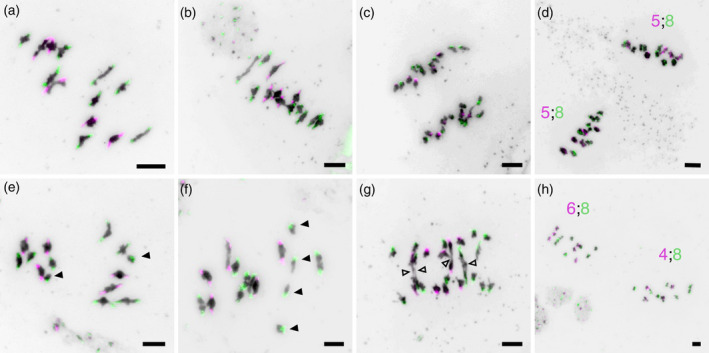
Fluorescence *in situ* hybridization (FISH) of centromeric repeats in *Arabidopsis suecica* meiocytes. Probes specific to the *Arabidopsis arenosa* (green) and *Arabidopsis thaliana* (pink) centromeric repeat sequences were hybridized to *A*. *suecica* meiocytes. (a–d) Representative cells showing normal error‐free metaphase I (a, b), anaphase I (c) and metaphase II (d). Errors were observed in a minority of cells, including univalents (filled triangles) at metaphase I (e, f), bridges (open triangles) at anaphase I (g) and aneuploidy at metaphase II (h). Coloured text in (d) and (h) indicates the number of chromosomes belonging to the *A*. *arenosa* (green) and *A*. *thaliana* (pink) subgenomes in the two cells following the first meiotic division. Scale bars: 5 μm. [Colour figure can be viewed at wileyonlinelibrary.com]

Meiotic progression in *A*. *suecica* was initially characterized using 4′,6‐diamidino‐2‐phenylindole (DAPI)‐stained meiotic chromosome spreads of developing floral buds (Figure 1). In the early prophase, meiosis progressed in a very similar manner to diploid *A*. *thaliana*, with full synapsis by pachytene (Figure 1c). At diakinesis, 13 bivalents were usually clearly visible (Figure 1e), although sometimes connections between bivalents persisted. At metaphase I 13 bivalents were also usually visible, although pairs of univalents were observed in approximately 10% of cells (Figures 1f; Figure S1, *n* = 9/87). No obvious multivalents were observed at metaphase I, although in some cells there were clusters of more than two chromosomes (Figure S1). From the DAPI‐stained images alone it was impossible to tell whether these represented overlapping bivalents or multivalents, although given the reduced spreading in these cells, our interpretation is that they are overlapping bivalents. The majority of anaphase‐I cells showed regular chromosome segregation, although bridges were sometimes observed (*n* = 4/30) (Figure 2; Figure S1). At metaphase II most cells contained 13 chromosomes (Figure 1j), with aneuploidy (12 or 14 chromosomes) observed in a minority of cells (*n* = 4/60) (Figure 2h). The aneuploidy observed at metaphase II is likely to result from the mis‐segregation of the univalents observed at metaphase I. This suggests that not all metaphase‐I univalents can be explained by the precocious splitting of bivalents.

### Fluorescent *in situ* hybridization (FISH) using subgenome‐specific centromeric probes suggests meiotic abnormalities are not caused by homoeologous recombination

To gain further insights into meiotic chromosome behaviour in *A*. *suecica* we performed FISH using probes able to distinguish the centromeric repeat sequences of the two subgenomes. At metaphase I all bivalents had pairs of centromeric signals from the same colour probe, with no instances of both probes hybridizing to the same bivalent (Figure 2a,b). Thus, we saw no evidence of non‐homologous recombination occurring between *A*. *arenosa* subgenome chromosomes and *A*. *thaliana* subgenome chromosomes. In addition, univalents always occurred in pairs, both hybridizing to the same probe (Figure 2e,f). No configurations with one trivalent and one univalent were observed. This is consistent with univalents arising via a lack of the obligate crossover, or premature loss of cohesion, rather than through homoeologous recombination.

Anaphase bridges could arise in *A*. *suecica* through homoeologous recombination which, given the different genomic structures of the *A*. *arenosa* and *A*. *thaliana* subgenomes, could lead to dicentric chromosomes containing one *A*. *thaliana* subgenome centromere and one *A*. *arenosa* subgenome centromere. However, all anaphase bridges observed always formed between two chromosomes hybridizing to the same centromeric probe (Figure 2g), and thus cannot be explained by homoeologous recombination. Clear evidence for aneuploidy at metaphase II was observed for both subgenomes. For example, Figure 2h shows two daughter cells following the first meiotic division, with one cell receiving four *A*. *thaliana* subgenome chromosomes and the other cell receiving six.

### Synapsis proceeds normally in *A*. *suecica*, with no evidence of synaptic partner switches

**Figure 3:**
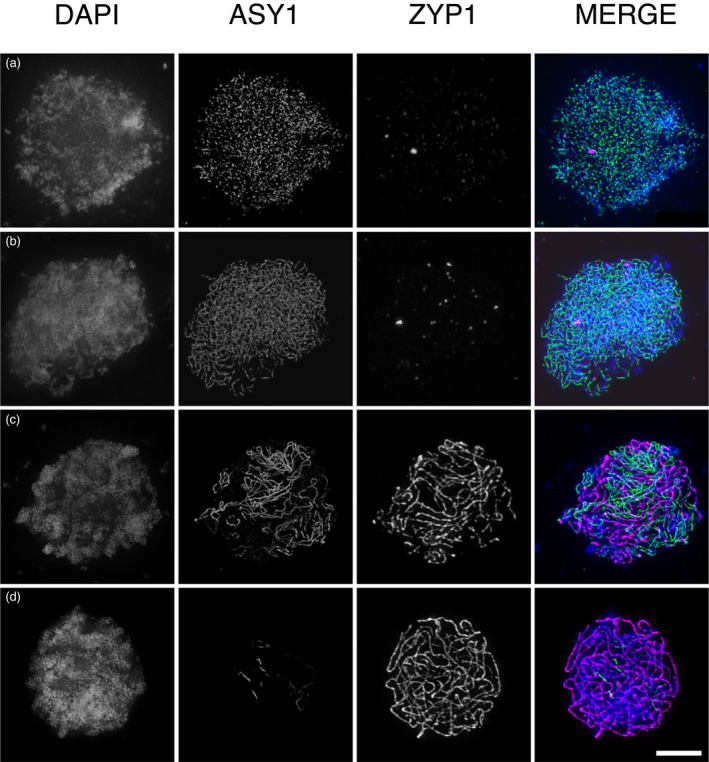
Progression of synapsis in *Arabidopsis suecica*. 3D‐SIM images showing the localization of ASY1 (green), ZYP1 (magenta) and DNA (DAPI, blue) in early meiotic prophase I. ASY1 first appears as distinct foci at G2/leptotene (a), progressing to fully extended axis by early late leptotene/early zygotene (b). ZYP1 first appears as distinct foci (a, b), with synapsis progressing throughout zygotene (c) and maximal synapsis achieved at pachytene (d). Scale bars: 5 μm. [Colour figure can be viewed at wileyonlinelibrary.com]

We used the immunolabelling of ASY1 and ZYP1 to monitor the progression of synapsis in *A*. *suecica* by three‐dimensional structured illumination microscopy (3D‐SIM). We detected loading of ASY1 in leptotene as discrete foci (Figure 3a), which extended to form the linear chromosome axes by the early zygotene (Figure 3b). Synapsis progressed through zygotene (Figure 3c), with the incorporation of ZYP1 and the specific unloading of ASY1 at synapsed axes. When maximal synapsis is achieved at the onset of pachytene (Figure 3d), ZYP1 labels all the chromatin whereas ASY1 only remains in a few very localized regions. The persisting regions of strong ASY1 signal may correspond to rDNA repeats, which tend to remain un‐synapsed at pachytene (Hurel et al., 2018; Sims et al., 2019). In autotetraploid *A*. *arenosa*, synaptic partner switches, observable by 3D‐SIM, are accompanied by persistent ASY1 labelling during the pachytene and late zygotene (Morgan et al., 2020; Morgan et al., 2021). These synaptic partner switches result in synaptic multivalents that may eventually lead to metaphase‐I multivalents (Morgan et al., 2020; Morgan et al., 2021). We analysed regions with persisting ASY1 labelling during late zygotene and pachytene. These regions were only associated with un‐synapsed axes and never had the typical structure of the synaptic partner switches observed in *A*. *arenosa* tetraploids (Morgan et al., 2020; Morgan et al., 2021). Although we saw no clear evidence of synaptic partner switches in *A*. *suecica* at the late zygotene through to the pachytene (*n* = 14; Figure 3c,d), we cannot rule out their occurrence at low frequency or earlier in the zygotene.

### Crossover number estimation in *A*. *suecica*

**Figure 4:**
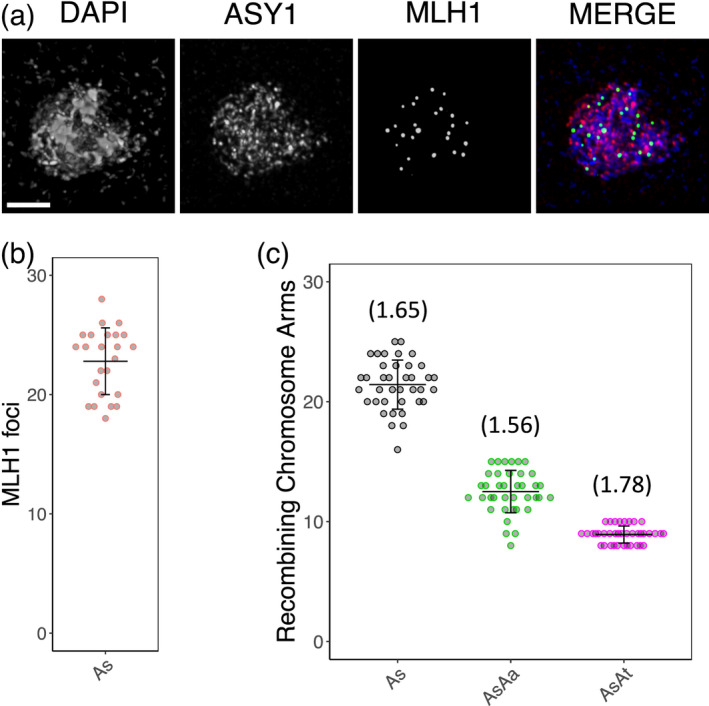
Crossover number estimation in *Arabidopsis suecica* (*As*). (a) Confocal image showing representative localization of DNA (DAPI, blue), ASY1 (red) and MLH1 (green) at diplotene. (b) The average number of MLH1 foci for *A*. *suecica* was determined, counting foci in cells at either diplotene or diakinesis stages (*n* = 24). (c) Crossovers numbers were also estimated by counting the number of recombining chromosome arms at metaphase I. Separate counts were established for the *Arabidopsis arenosa* (*AsAa*, green) and *Arabidopsis thaliana* (*AsAt*, magenta) subgenome chromosomes by analysing FISH images such as those shown in Figure 2. Numbers in parentheses indicate the average number of recombining arms per chromosome. Scale bar: 5 μm. [Colour figure can be viewed at wileyonlinelibrary.com]

It has previously been reported that newly formed synthetic Arabidopsis polyploids have increased levels of recombination (Pecinka et al., 2011). In newly formed autotetraploid *A*. *arenosa*, recombination is more than doubled compared with the diploid (diploid = 10 HEI10 foci, neoteraploid = 24 HEI10 foci; Morgan et al., 2021). However, naturally evolved autotetraploid accessions have a reduced recombination of around one crossover per chromosome pair (17 HEI10 foci or 1.06 per chromosome pair), which is likely to be associated with meiotic adaptation (Morgan et al., 2020; Morgan et al., 2021). We used immunolocalization of MLH1 to determine the number of class‐I crossovers in *A*. *suecica* (Figure 4). We observed an average of 22.8 MLH1 foci (1.75 crossovers per chromosome pair), similar to the number expected given the numbers of class‐I crossovers described for the diploid progenitors *A*. *arenosa* (approx. 10 HEI10 foci; Morgan et al., 2021) and *A*. *thaliana* (approx. 7–10 MLH1 foci, depending on the ecotype; e.g. Capilla‐Pérez et al., 2021) (Figure 4). To confirm that sites marked with MLH1 foci do go on to become true crossovers, we also estimated crossover number by counting the number of chromosome arms with at least one chiasma in well‐spread metaphase‐I cells (*n* = 38; Figure 4c). As the number of arms with crossovers cannot be more than two, these counts slightly underestimate crossover number. Given the expected slight underestimation, the chiasma counts were in strong agreement with MLH1 foci numbers (Figure 4c). Using FISH (Figure 2a) we could also separately count the chromosome arms with crossovers for each subgenome. As expected, given their larger average physical size and genetic map length (Burns et al., 2021), *A*. *thaliana* subgenome chromosomes had more recombining arms on average than *A*. *arenosa* subgenome chromosomes (1.78 vs 1.56, *P* = 1.05E‐6, paired Student's *t*‐test).

### Diploid alleles of meiotic genes under strong selection in autotetraploid *A*. *arenosa* are found in the *A*. *arenosa* subgenome of *A*. *suecica*

**Figure 5:**
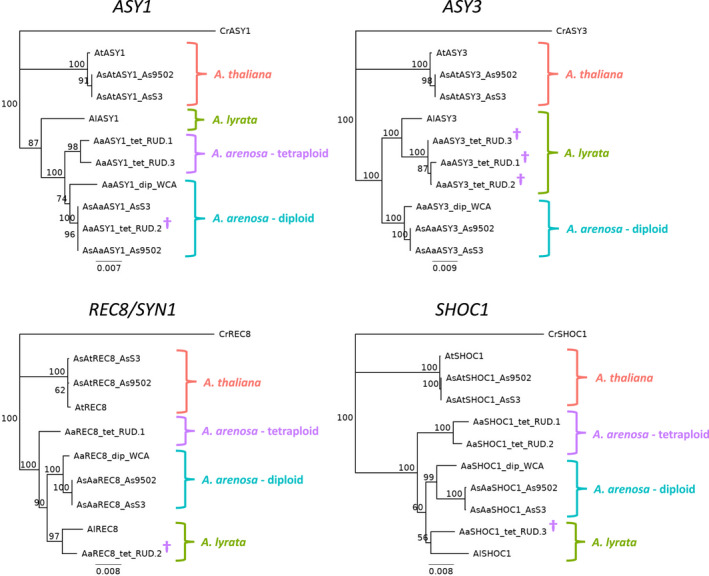
Phylogenetic trees of *ASY1*, *ASY3*, *REC8/SYN1* and *SHOC1*. Phylogenetic trees are based on aligned coding sequences of *Arabidopsis thaliana* (*At*), diploid *Arabidopsis arenosa* (Aa–dip), tetraploid *A*. *arenosa* (Aa–tet), *Arabidopsis lyrata* (Al) and the two *Arabidopsis suecica* subgenomes (AsAa and AsAt) with *Capsella rubella* (Cr) as an outgroup. AsAt subgenome sequences consistently clustered with *A*. *thaliana*. AsAa subgenome sequences consistently clustered with diploid *A*. *arenosa*. Some tetraploid *A*. *arenosa* alleles clustered as an out‐group to diploid *A*. *arenosa*, whereas other alleles (†) clustered with *A*. *lyrata* (e.g. *ASY3*) or with diploid *A*. *arenosa* (e.g. *ASY1*), reflecting extensive interploidy and interspecific admixture. For *A*. *suecica*, sequences of both the AsS3 and As9502 assemblies are included. The diploid *A*. *arenosa* assembly comes from a western Carpathian population (WCA) from Slovakia and the tetraploid assembly comes from a ruderal (RUD) population in Sweden. For each gene, multiple alleles are present within the assembly of the tetraploid *A*. *arenosa* individual, all of which are included in the final phylogenies. Bootstrap confidence levels (percentage of 1000 replicates) are indicated at each node. [Colour figure can be viewed at wileyonlinelibrary.com]

A number of genes associated with the chromosome axis (e.g. *ASY1*, *ASY3*, *PRD3*, *PDS5B*, *SYN1/REC8*, *SHOC1* and *ZYP1b*) show strong signatures of selective sweeps in autotetraploid *A*. *arenosa* (Bohutínská et al., 2021; Wright et al., 2015; Yant et al., 2013). Given that the *A*. *arenosa* subgenome of *A*. *suecica* is hypothesized to have been contributed by an autotetraploid donor (Novikova et al., 2017), we looked to see whether the tetraploid‐specific variants of these meiotic genes were also found in allopolyploid *A*. *suecica*. For this comparison we looked first at the diploid/tetraploid variants predicted to affect protein structure or function identified by Bohutínská et al. (2021), restricting our analysis to ploidy‐specific variants that we defined as sites where the non‐reference allele was the predominant variant in one cytotype (i.e. allele frequency > 50%) and was present at less than 5% in the other cytotype. Across these 87 sites, the *A*. *arenosa* subgenome of the two *A*. *suecica* reference sequences (Burns et al., 2021; Jiang et al., 2021) had the diploid variant at 83 sites, the tetraploid variant at no sites and an alternative variant at four sites (Table 1 and Table S1). For several of these genes we retrieved full‐length sequences from the draft diploid (GCA_905216605.1) and tetraploid (GCA_905175405.1) *A*. *arenosa* assemblies to generate phylogenetic trees (Figure 5). The diploid *A*. *arenosa* assembly is of an individual from a Western Carpathian population in Strečno, Slovakia, and the tetraploid *A*. *arenosa* assembly is of an individual from a ruderal population in Sweden (Burns et al., 2021). For the highly heterozygous tetraploid individual, each gene is present on multiple scaffolds representing different alleles present in the individual sequenced. For all genes, the sequences of the *A*. *arenosa* subgenome of *A*. *suecica* (*AsAa*) clustered with the diploid *A*. *arenosa* sequence (Figure 5). The tetraploid *A*. *arenosa* alleles showed different relationships for different genes, with some alleles clustering independently, some alleles clustering with *A*. *lyrata* and some alleles clustering with diploid *A*. *arenosa*, demonstrating the extensive interploidy and interspecific admixture previously reported for *A*. *arenosa* and *A*. *lyrata* (Seear et al., 2020; Monnahan et al., 2019; Arnold et al., 2015). A clear example of this is *ASY3*, where all tetraploid *A*. *arenosa* alleles clustered with *A*. *lyrate*, reflecting the previously described recent introgression of an *ASY3* allele from *A*. *lyrata* (Seear et al., 2020).

**Table 1:** Ploidy‐specific *Arabidopsis arenosa* meiotic gene variants found in *Arabidopsis sucecica* ^a^

Variant type	AsS3 (Burns et al., 2021)	As9502 (Jiang et al., 2021)
Diploid	83	83
Tetraploid	0	0
Alternate	4	4
Total sites	87	87

^a^ Meiotic gene variants predicted to affect protein function from Bohutínská et al. (2021).

### The *A*. *arenosa* subgenome of *A*. *suecica* has a high percentage of derived meiotic gene variants found in diploid Baltic and south‐eastern Carpathian lineages

In addition to meiotic genes showing strong signatures of selection in autotetraploid *A*. *arenosa*, a number of meiotic genes, including *REC8*, *ASY3*, *SMG7*, *PDS5e*, *SMC6b* and *RMI1*, show signatures of selection in specific diploid lineages (Bohutínská et al., 2021; Wright et al., 2015). Among the 56 variants identified by Bohutínská et al. (2021) that are predicted to affect protein structure or function and differ between diploid populations, *A*. *suecica* has 24 of the 30 derived variants found in Baltic and closely related south‐eastern Carpathian populations and two of the 26 derived variants found in other diploid populations (Table 2).

**Table 2:** Derived diploid *Arabidopsis arenosa* gene variants found in *Arabidopsis sucecica*

Diploid population	Number of derived variants^a^	AsS3 (Burns et al., 2021)			As9502 (Jiang et al., 2021)		
		Ancestral	Derived	Alternate	Ancestral	Derived	Alternate
Baltic	19	2	16	1	2	16	1
Dinaric	10	8	2	0	8	2	0
Pannonian	16	15	0	1	15	0	1
South‐eastern Carpathian	11	2	8	1	2	8	1
Western Carpathian	0	0	0	0	0	0	0

^a^ Meiotic gene variants predicted to affect protein function in Bohutínská et al. (2021).

## DISCUSSION

Meiotic recombination and accurate chromosome segregation are challenging processes for polyploid organisms, and the nature of these challenges differ between autopolyploids and allopolyploids (Lloyd & Bomblies, 2016). The *Arabidopsis* genus, which includes multiple diploid, autotetraploid and allotetraploid species, provides a useful system to interrogate these different meiotic challenges. We have presented here a description of meiosis in wild‐type allopolyploid *A*. *suecica* that will serve as a reference example of allopolyploid meiosis and a useful companion to previous characterizations of meiosis in diploid *A*. *thaliana* (Armstrong & Jones, 2003; Hurel et al., 2018; Prusicki et al., 2019; Ross et al., 1996) and autotetraploid *A*. *arenosa* (Higgins et al., 2014; Morgan et al., 2021).

In general, we observed that meiosis in *A*. *suecica* was largely diploid‐like, with chromosomes able to undergo complete synapsis by the pachytene. In autotetraploid *A*. *arenosa*, where each chromosome is present in four equally divergent copies (homologues), chromosomes form synaptic associations with more than one partner chromosome, resulting in synaptic partner switches at the pachytene (Morgan et al., 2021). We found no clear evidence for such partner switches in allotetraploid *A*. *suecica*, suggesting that synapsis only occurs between true homologues or that any early homoeologous synapsis is corrected by the mid to late zygotene, such as is seen in *Brassica napus* (Grandont et al., 2014). Unsurprisingly, therefore, metaphase‐I bivalents always formed between homologous chromosomes and there was no multivalent formation, indicating the complete suppression of homoeologous crossovers. Despite a largely regular diploid‐like meiosis, we did observe some low‐frequency errors in *A*. *suecica* meiosis, including univalent formation at metaphase I, anaphase bridges and aneuploidy at metaphase II. Univalents at metaphase I and anaphase bridges could result from the asynchronous loss of cohesion, particularly in some cases where homologous univalents are observed directly opposite one another across the metaphase plate or where homologous bivalents remain intact during the early anaphase. However, the aneuploidy observed at metaphase II indicates segregation errors, suggesting that at least some metaphase‐I univalent formation is caused by a lack of complete crossover assurance.

Although we see some univalents at metaphase I, both allopolyploidy and autopolyploidy have previously been associated with an immediate increase in recombination. Using a fluorescent seed‐based system, Pecinka et al. (2011) surveyed one genetic interval and observed increased recombination in neoautotetraploid *A*. *thaliana* and neoallotetraploid *A*. *suecica*, compared with the same interval in diploid *A*. *thaliana*. A similar genome‐wide increase is observed in neoautotetraploid *A*. *arenosa*, which have approximately 24 HEI10 foci at pachytene (1.5 per chromosome pair), more than double the number observed in the diploid (approx. 10 HEI10 foci or 1.25 per chromosome pair; Morgan et al., 2021). Despite this immediate increase in recombination following polyploidization, established autotetraploid populations show remarkably low crossover numbers, with around 17 HEI10 foci per cell, or 1.06 per chromosome pair, and thus almost exclusively form bivalents at metaphase I (Morgan et al., 2021). By default, restricting each chromosome to only one crossover event ensures strict bivalent formation even in the complete absence of partner preference. The reduction in crossover number in tetraploid *A*. *arenosa* is accompanied by strong signatures of selection in genes associated with the meiotic chromosome axis and the synaptonemal complex, including *ASY3*, *ASY1*, *ZYP1*, *REC8*, *PDS5* and *PRD3* (Bohutínská et al., 2021; Wright et al., 2015; Yant et al., 2013). Tetraploid variants of these genes are thought to act together to reduce crossover number through ‘supercharged’ crossover interference (Morgan et al., 2020; Morgan et al., 2021). Interestingly, it is also speculated that introgression of these tetraploid alleles may have aided the establishment of autotetraploid *A*. *lyrata* populations (Seear et al., 2020), presumably via the same mechanism.

Although reducing crossover number is a successful strategy to stabilize meiosis in autotetraploid Arabidopsis, it is clearly not the route taken in allopolyploid *A*. *suecica*, as we observed an average of around 1.75 crossovers per chromosome pair. Furthermore, we show that *A*. *suecica* has diploid‐like variants of the genes under strong selection in autotetraploid *A*. *arenosa*. This is despite previous reports that an autotetraploid individual was the donor of the *A*. *arenosa* subgenome to *A*. *suecica* (Novikova et al., 2017). Diploid‐like variants in *A*. *suecica* could arise in several ways. One possibility is that the donor of the *A*. *arenosa* subgenome to *A*. *suecica* was in fact a diploid, and potentially an ancestor of modern‐day Baltic populations, with previous interpretations of a tetraploid donor (Novikova et al., 2017) confounded by the extensive interploidy gene flow that occurs between diploid and autotetraploid *A*. *arenosa* populations in northern Europe (Monnahan et al., 2019). Similarly, interploidy gene flow in *A*. *arenosa* may have provided a route for diploid‐like alleles into *A*. *suecica*, even if the original donor of the *A*. *arenosa* subgenome was tetraploid. An alternative hypothesis, given similar age estimates for autotetraploid *A*. *arenosa* and allotetraploid *A*. *suecica* (Arnold et al., 2015; Bohutínská et al., 2021; Burns et al., 2021; Novikova et al., 2017), is that the *A*. *arenosa* subgenome of *A*. *suecica* represents a very early tetraploid *A*. *arenosa* genome. If this were the case, the donation of the *A*. *arenosa* subgenome to *A*. *suecica* would have had to occur before the major component of tetraploid *A*. *arenosa* meiotic adaptation associated with *de novo* mutations took place (Bohutínská et al., 2021). Although more detailed population genetic analyses will be required to distinguish between these (or other) scenarios, our results do cast some doubt on the hypothesized tetraploid origin of the *A*. *arenosa* subgenome.

Regardless of their origin, the presence of diploid‐like alleles of key meiotic genes in *A*. *suecica* suggests that these genes have different selective constraints in autopolyploids and allopolyploids, and that autopolyploid *A*. *arenosa* and allopolyploid *A*. *suecica* have followed different evolutionary trajectories to stabilize their meiotic behaviour. This difference in evolutionary trajectory is unsurprising given the different challenges posed by allopolyploid and autopolyploid meiosis. In allopolyploids, limiting the crossover number is not necessarily a route to meiotic stability. The requirement to ensure recombination only occurs between homologous chromosomes and not homoeologous chromosomes, meaning that the stringency of crossover formation rather than crossover number is likely to be of primary significance.

Despite some low‐frequency meiotic errors, naturally evolved populations of *A*. *suecica* have vastly improved fertility and meiotic outcomes compared with first‐generation neopolyploid *A*. *suecica* (Henry et al., 2014). The *BYS* QTL identified by Henry et al. (2014) on *A*. *suecica* chromosome 9 (chromosome 4 of the *A*. *arenosa* subgenome) explains approximately 10% of this variation in meiotic stability; however, the gene underlying the QTL is unknown. Possible candidates are genes in the mismatch repair pathway, which is implicated in the suppression of homoeologous crossovers through the detection of mismatches in the recombination intermediate heteroduplex (Bozdag et al., 2021; Serra et al., 2021; Spies & Fishel, 2015; Sugawara et al., 2004). Other candidates are genes encoding the ZMM proteins MSH4 and ZIP4, which promote recombination intermediate stability and have been shown to influence the formation of homoeologous crossovers in *Brassica* (Gonzalo et al., 2019) and wheat (Martín et al., 2021; Rey et al., 2017), respectively. None of these obvious candidates are within the *BYS* QTL, however. Future comparative studies of meiosis in evolved and synthetic *A*. *suecica*, as well as studies of CRISPR‐induced mutants of key candidate genes, will therefore be needed to further our understanding of crossover stringency and meiotic adaptation in allopolyploid *A*. *suecica*.

## EXPERIMENTAL PROCEDURES

### Plant materials

*Arabidopsis suecica* seeds were originally obtained from Jeffrey Chen at The University of Texas at Austin, USA. Our laboratory strain (JC2) was derived from a single plant presumed to be of accession As9052. To confirm, we compared the relatedness of different *A*. *suecica* accessions using available whole‐genome resequencing data. As expected, principal component analysis (PCA) confirmed that JC2 was most closely related to As9502 (Figure S2). As9502 is derived from accession 90‐10‐085‐10, originally collected in Finland and then cultivated for several decades in laboratory environments (Pontes et al., 2003). It is one of two strains recently used to generate *de novo* genome assemblies (Jiang et al., 2021).

Plants were initially grown in long‐day conditions (16‐h light/ 8‐h dark cycle) in growth chambers or glasshouses at 21°C. After 4 weeks plants were moved to long‐day conditions at 4°C for a minimum of 4 weeks for vernalization before returning to growth chambers at 21°C to induce flowering. Chromosome numbers of all plants were validated prior to use in cytological assays to ensure that they were euploid (i.e. 2*n* = 26; e.g. Figure 2l).

### DAPI spreads

DAPI spreads were undertaken as described by Chelysheva et al. (2010)), with minor modifications. Inflorescences were fixed for a minimum of 3 days in Carnoy's fixative (3:1 ethanol:acetic acid, v/v), with several changes of fixative in the first 24 h. Fixed inflorescences were rinsed in water (once) and 10 mm citrate buffer, pH 4.5 (twice), then single buds of the desired size (0.3–1.0 mm) were placed in individual wells of a flat‐bottom 96‐well plate containing 50 μL of citrate buffer. Citrate buffer was replaced with 50 μL of digestion mix (0.3% w/v cellulase RS, 0.3% w/v pectolyase Y23, 0.3% w/v cytohelicase in citrate buffer) and digested at 37°C for 2.5–3.0 hours. After digestion, the digest mix was removed from the well, replaced with water and the plate kept on ice. Single buds were placed in 4 μL of water on a clean slide and tapped with a brass rod to transform into a cell suspension. Then, 12.5 μL of 80% acetic acid was added to the cells, the slides placed on a 45°C heat block and cells stirred continuously with a hook for 2 min. After the first minute another 12.5 μL of 80% acetic acid was added. Cells were fixed on slides by pipetting Carnoy's fixative around the drop of cleared cell suspension and air‐dried. Slides were then mounted in 7 μL of VECTASHIELD (Vector Laboratories, https://vectorlabs.com) with 2 μg mL^–1^ DAPI.

### Fluorescence *in situ* hybridization (FISH)

A Rhodamine‐labelled centromeric repeat probe for FISH was prepared from *A*. *thaliana* genomic DNA (Col‐0) by polymerase chain reaction (PCR) using primers AL190, 5′‐AGTCTTTGGCTTTGTGTCTT‐3′ (AlU; Kawabe & Nasuda, 2005), and AL205, 5′‐TGGACTTTGGCTACACCATC‐3′, with sequences labelled with 40 μMm tetramethyl‐rhodamine‐5‐dUTP (Roche, https://www.roche.com) by PCR. A fluorescein‐labelled centromeric probe was prepared from *A*. *arenosa* genomic DNA (SNO) by PCR using primers AL203, 5′‐AGTTTTCGGTTTTGGAGCTT‐3′, and AL204, 5′‐AGGACTTCGGCCACACCCAC‐3′ (AaU and AaR, Kawabe & Nasuda, 2005), with 0.1 mm Fluorescein‐12‐dUTP (ThermoFisher Scientific, https://www.thermofisher.com). Slides were prepared as described for the DAPI spreads then denatured using 40 μL of 70% deionized formamide (Sigma‐Aldrich, https://www.sigmaaldrich.com) under a parafilm coverslip for 15 min at 65°C in a humid chamber. Slides were dehydrated for 2 min successively in 70%, 90% and 100% ethanol, and then air‐dried. A 40‐μL volume of hybridization mix (50% deionized formamide, 2× SSC, 10% dextran sulphate, 0.5 mg mL^−1^ sonicated salmon sperm DNA and aprox. 100 ng of each probe; all reagents supplied by Sigma‐Aldrich) was denatured for 5 min at 90°C, added to the slide and a parafilm coverslip floated on top. Slides were then incubated overnight at 37°C in a humid chamber. After incubation slides were washed in 2× SSC for 5 min at room temperature, 0.2× SSC for 10 min at 60°C, 5 min in phosphate‐buffered saline with 0.1% Triton X‐100 (PBS‐T; Sigma‐Aldrich) at room temperature (20‐25°C) and 5 min in PBS at room temperature. Finally, slides were dehydrated as described above and mounted with 7 μL of Vectashield with 2 μg mL^−1^ DAPI.

### Immunolabelling for SIM

Immunolabelling of *A*. *suecica* (JC2) meiocytes for SIM was undertaken as previously described for *A*. *arenosa* (Morgan & Wegel, 2020). Briefly, anthers containing meiocytes of the desired meiotic stage were dissected from four to six fresh buds and macerated on a No. 1.5H coverslip (Marienfeld, https://www.marienfeld‐superior.com) in 5 μL of digestion medium (0.4% cytohelicase, 1.5% sucrose, 1% polyvinylpyrrolidone (MW 40000) in sterile water; Sigma‐Aldrich) for 1 min using a brass rod. Coverslips were then incubated in a moist chamber at 37°C for 5 min before adding 10 μL of 2% Lipsol solution (SciLabware, now DWK Life Sciences, https://www.dwkltd.com) and macerating with a brass rod for 2 min. A 20‐μL volume of 4% paraformaldehyde (pH 8) was then added and allowed to air‐dry for 3 h. Coverslips were then blocked in 0.3% bovine serum albumin in PBS‐T and then incubated with primary antibody overnight at 4°C. Coverslips were washed three times for 5 min in 1× PBS‐T before incubating with secondary antibody for 2 h at 37°C. Coverslips were finally incubated in 10 μg mL^−1^ DAPI for 5 min then washed three times in 1× PBS‐T and once in sterile water before being mounted on a slide in 7 μL of Vectashield (Vector Laboratories). The antibodies used were α‐ASY1 (rat, 1:500; Armstrong et al., 2002) and α‐ZYP1 (guinea pig, 1:500; Higgins et al., 2005), with secondary antibodies (Alexa Fluor 488 chicken anti‐rat, A21470, and Alexa Fluor 633 goat anti‐guinea pig, A21105; Invitrogen, now ThermoFisher Scientific) used at 1:500 dilution. Immunostained cells were imaged using structured illumination microscopy (3D‐SIM) on a Zeiss Elyra PS1 microscope (https://www.zeiss.com) and images analysed in fiji (https://imagej.net/software/fiji). Diplotene and diakinesis cells were identified by the lack of ZYP1 and the presence of MLH1 foci.

### MLH1 immunolabelling

Meiocytes from *A*. *suecica* (JC2) were embedded in acrylamide to preserve their three‐dimensional structure and then used for the immunolocalization studies, as previously described (Nibau et al., 2020; Phillips et al., 2010). Briefly, young buds (<1 mm) were harvested and fixed in 2% (w/v) paraformaldehyde, washed, macerated with a brass rod in 1% (v/v) Lipsol in buffer A and embedded in acrylamide. Embedded meiocytes were blocked and incubated with α‐ASY1 (rat, 1:500; Armstrong et al., 2002), α‐ZYP1 (guinea pig, 1:500; Higgins et al., 2005) and α‐MLH1 (1:250; Chelysheva et al., 2010) antibody solution for 24–36 h. After washing, embedded meiocytes were incubated overnight with secondary antibodies (Alexa Flour 568 goat anti‐rat, A11077, Alexa Fluor 488 goat anti‐rabbit, A32731, and Alexa Fluor 633 goat anti‐guinea pig, A21105; Invitrogen, now ThermoFisher Scientific), used at 1:500 dilution. Images were acquired using a Leica TCS SP8 confocal microscope (https://www.leica‐microsystems.com) with maximum projection of *Z*‐stacks and deconvolved using the built‐in lightning software. Image analysis was carried out in imaris 7.3 (Oxford Instruments, https://imaris.oxinst.com). Diakinesis and diplotene cells were identified for image acquisition based on the presence of ASY1 signal and MLH1 foci and the absence of ZYP1 signal.

### Sequencing

For lab strain JC2, DNA was extracted from leaf tissue according to a cetyl trimethylammonium bromide (CTAB) protocol (Doyle & Doyle, 1987), with minor alterations and the inclusion of a sorbitol pre‐wash step (Inglis et al., 2018). Briefly, approximately 200 mg of leaf tissue was ground in a 1.5‐mL tube with 1 mL of sorbitol wash solution (100 mm Tris–HCl, pH 8.0, 0.35 m d‐sorbitol, 5 mm EDTA, 1% polyvinylpyrrolidone (MW 40000) and 1% b‐mercaptoethanol; Sigma‐Aldrich), then centrifuged at 6000 ***g*** for 5 min. The supernatant was discarded and the pellet resuspended in 500 μL of extraction buffer (100 mm Tris–HCl, pH 8.0, 2% CTAB, 1.4 m NaCl, 0.5 m EDTA and 1% b‐mercaptoethanol; Sigma‐Aldrich), then incubated at 60°C for 1 h. The sample was briefly centrifuged to pellet solids and 400 μL of liquid phase transferred to a new tube before being extracted twice in an equal volume of 24:1 chloroform:isoamylalcohol. Between the first and second chloroform:isoamylalcohol extractions, 0.5 μL of RNase Cocktail Enzyme Mix (Invitrogen, now ThermoFisher Scientific) was added and the sample incubated at 37°C for 2 h. DNA was precipitated in an equal volume of isopropanol at −20°C for 30 min before centrifugation to pellet DNA. The pellet was washed first in 76% ethanol, 0.2 m sodium acetate and then 76% ethanol, before air‐drying. DNA was resuspended in 50 μL of TE‐buffer for storage, and small fragments were removed using Agencourt AMPure XP beads (Beckman Coulter, https://www.beckmancoulter.com) following the manufacturer's instructions, with a DNA:beads ratio of 0.4. We quantified the extracted gDNA using the dsDNA HS assay (Q32854) from ThermoFisher Scientific with their Qubit 2.0 or 3.0 (Q33216). We prepared TruSeq PCR‐free (FC‐121‐3003) sequencing libraries for a 350‐bp insert length of genomic DNA using 500 ng of DNA as the input. Short‐read data for JC2 are available on the Sequence Read Archive under Bioproject PRJNA819005.

### Variant analysis for meiotic genes

*Arabidopsis lyrata* sequences for genes identified by Bohutínská et al. (2021)) were downloaded from Plaza Dicots 4.5 and used to identify *A*. *suecica* orthologues for the two reference genomes using BLAST within geneious prime 2020.0.5. Multispecies protein alignments (also including *A*. *thaliana*) were generated using geneious prime and manually interrogated to identify the variant on the *A*. *arenosa* subgenome of the respective *A*. *suecica* reference genomes. We restricted our analysis to ploidy‐specific variants that we defined as sites where the non‐reference allele was the predominant variant in one cytotype (i.e. with an allele frequency of >50%) and was present at less than 5% in the other cytotype. The raw allele counts from Bohutínská et al. (2021)) were kindly provided by Pirita Paajanen.

### Variant analysis for PCA

Our data‐processing pipeline involved three main parts: (i) preparing the raw sequencing data; (ii) mapping the sequencing data; and (iii) variant discovery (gatk 4.2.4.1, following gatk best practices). Fastq.gz files for all accessions except JC2 were downloaded from the short‐read archive with sra‐toolkit (for all accession data, see Table S2). Reads were mapped to the AsS3 *A*. *suecica* reference genome (GCA_905175345.1) using bwa‐mem 2. We removed duplicate reads using ‘MarkDuplicates’ from picard tools (gatk 4.2.4.1), followed by ‘AddOrReplaceReadGroups’ to add read groups and indices to the .bam files. Our final data set for analysis contained 17 *A*. *suecica* individuals from three countries of origin. We called variants for the 17 .bam files using ‘HaplotypeCaller’ and ‘GenotypeGVCFs’ (gatk 4.2.4.1). We combined the single‐sample GVCF output from HaplotypeCaller to multisample GVCFs and then ran ‘GenotypeGVCFs’. Using ‘SelectVariants’ in gatk, we removed sites that had excess read depth, defined as 2 × modal read depth (DP > 1330). We inferred relationships among the *A*. *suecica* population based on single‐nucleotide polymorphisms (SNPs) using PCA. We used plink2 2.00a2.3LM for the analysis, first performing linkage pruning before running the PCA analysis. The resulting eigenvectors were then plotted with ggplot2 (r).

### Phylogenetic analyses

Gene sequences were retrieved for *A*. *thaliana* (TAIR10.1), *A*. *lyrata* (GCA_000004255.1), diploid *A*. *arenosa* (GCA_905216605.1), tetraploid *A*. *arenosa* (GCA_905175405.1), *A*. *suecica* (AsS3, GCA_905175345.1; As9502, GCA_019202805.1) and *Capsella rubella* (GCA_000375325.1) assemblies using BLAST. For tetraploid *A*. *arenosa*, genes were annotated using geneious prime 2020.0.5. Coding sequence alignments and neighbour‐joining trees were generated and plotted using geneious prime, with default settings.

## AUTHOR CONTRIBUTIONS

CN and AL designed the research. CN, AG, AE, WS‐J and AL performed the research. CN, DP and AL analysed the data. CN and AL wrote the article.

## CONFLICT OF INTEREST

All authors declare that they have no financial or non‐financial conflicts of interest in the subject matter or materials discussed in this article.

## Supporting information


**Figure S1** Representative DAPI‐stained metaphase‐I and anaphase‐I meiocytes.

**Figure S2** Principal component analysis (PCA) of *A*. *suecica* accessions.

**Table S1** *Arabidopsis suecica* variant data for meiotic genes under selection in *A. arenosa*.

**Table S2** Accession details for *Arabidopsis suecica* short‐read sequencing data.

## Data Availability

All Microscopy images are available at https://doi.org/10.20391/107b344b‐d0d0‐450f‐b24f‐dcfd2131b640. Short‐read sequencing data are available under the NCBI BioProject PRJNA819005.
