# Supplementary figures and images for: Meiosis in allopolyploid Arabidopsis suecica
Source: Plant J. 2022 Jul 22;111(4):1110–22. doi: 10.1111/tpj.15879 (PMC9545853; doi:10.1111/tpj.15879)

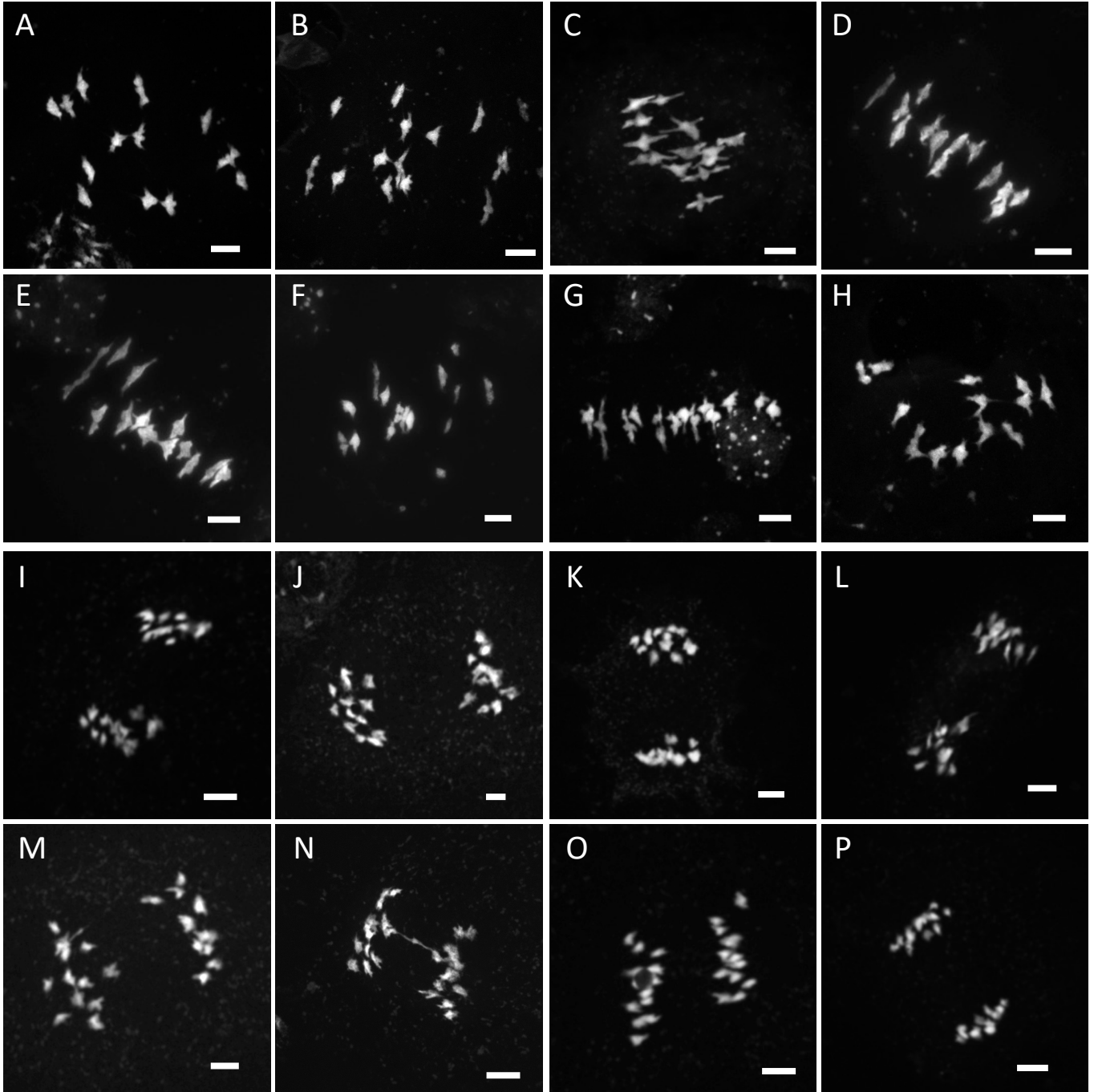

Supplement: Supplementary file 1 — Figure S1 Representative DAPI‐stained metaphase‐I and anaphase‐I meiocytes. [file TPJ-111-1110-s004.pdf]

ASuecica-PCA

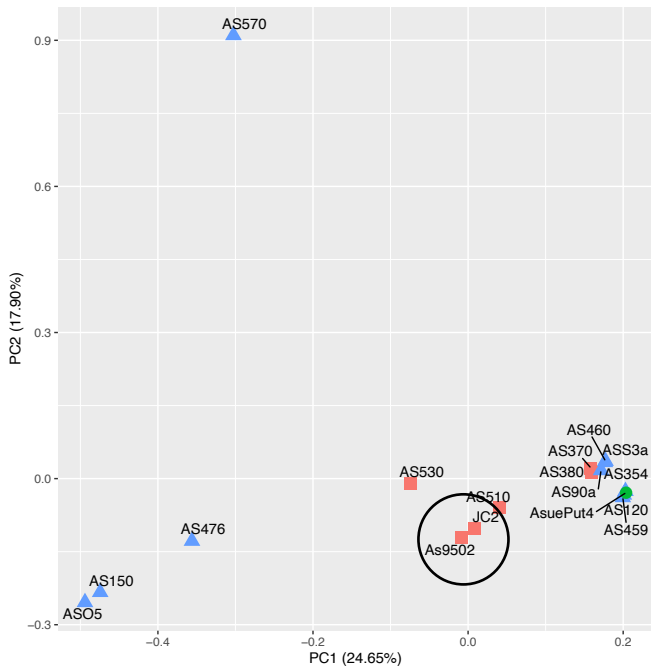

ASuecica-PCA

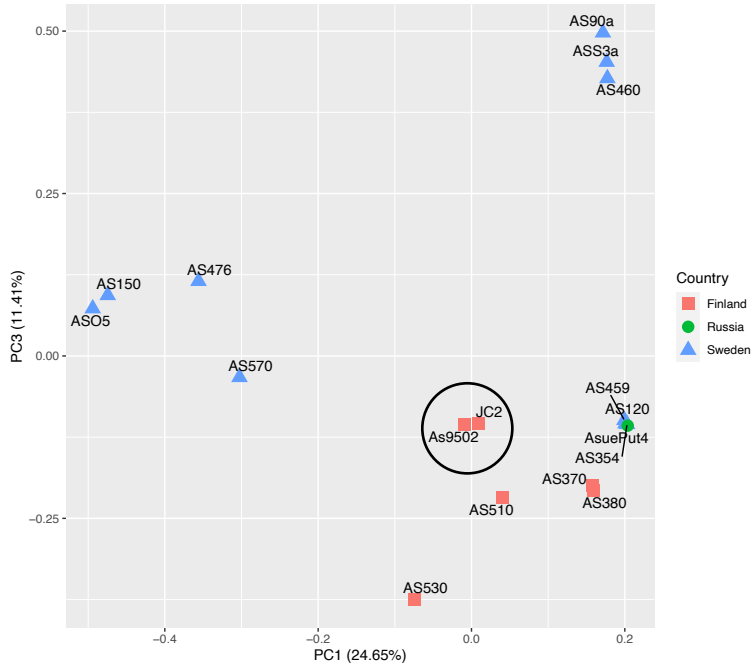

Supplement: Supplementary file 2 — Figure S2 Principal component analysis (PCA) of A. suecica accessions. [file TPJ-111-1110-s001.pdf]
